# Population differentiation or species formation across the Indian and the Pacific Oceans? An example from the brooding marine hydrozoan *Macrorhynchia phoenicea*

**DOI:** 10.1002/ece3.3236

**Published:** 2017-09-06

**Authors:** Bautisse Postaire, Pauline Gélin, J. Henrich Bruggemann, Marine Pratlong, Hélène Magalon

**Affiliations:** ^1^ UMR ENTROPIE Université de La Réunion/CNRS/IRD Université de La Réunion Saint Denis France; ^2^ Laboratoire d'Excellence CORAIL Perpignan France; ^3^ IMBE UMR 7263 Aix Marseille Université/CNRS/IRD/Avignon Université Marseille France; ^4^ I2M Equipe Evolution Biologique et Modélisation Aix Marseille Université/CNRS/Centrale Marseille Marseille France

**Keywords:** assignment test, brooding species, cryptic diversity, Hydrozoa, microsatellites

## Abstract

Assessing population connectivity is necessary to construct effective marine protected areas. This connectivity depends, among other parameters, inherently on species dispersal capacities. Isolation by distance (IBD) is one of the main modes of differentiation in marine species, above all in species presenting low dispersal abilities. This study reports the genetic structuring in the tropical hydrozoan *Macrorhynchia phoenicea* α (*sensu* Postaire *et al*., 2016a), a brooding species, from 30 sampling sites in the Western Indian Ocean and the Tropical Southwestern Pacific, using 15 microsatellite loci. At the local scale, genet dispersal relied on asexual propagation at short distance, which was not found at larger scales. Considering one representative per clone, significant positive *F*_*IS*_ values (from −0.327*** to 0.411***) were found within almost all sites. Gene flow was extremely low at all spatial scales, among sites within islands (<10 km distance) and among islands (100 to >11,000 km distance), with significant pairwise *F*_*ST*_ values (from 0.035*** to 0.645***). A general pattern of IBD was found at the Indo‐Pacific scale, but also within ecoregions in the Western Indian Ocean province. Clustering and network analyses identified each island as a potential independent population, while analysis of molecular variance indicated that population genetic differentiation was significant at small (within island) and intermediate (among islands within province) spatial scales. As shown by this species, a brooding life cycle might be corollary of the high population differentiation found in some coastal marine species, thwarting regular dispersal at distances more than a few kilometers and probably leading to high cryptic diversity, each island housing independent evolutionary lineages.

## 1 INTRODUCTION

In the context of biodiversity loss (Kolbert, 2014; Myers, Mittermeier, Mittermeier, da Fonseca, & Kent, 2000), assessing the degree of genetic connectivity (i.e., effective dispersal with consequences on gene flow; Carpenter et al., 2011; Jones et al., 2009; Schiavina, Marino, Zane, & Melià, 2014; Vellend & Geber, 2005) of marine populations is essential to establish effective marine protected areas. Such knowledge is valuable for determining the appropriate geographic span of their networks (Gerber et al., 2003), assuring the conservation of both evolutionary processes and alpha diversity (Christie et al., 2010). It is also indispensable for delineating relatively isolated populations or groups of populations more sensible to environmental variations, as they lack the capacity to acquire genetic variability from other populations (Cowen, Gawarkiewicz, Pineda, Thorrold, & Werner, 2007; Hellberg, 2009). Furthermore, connectivity studies can help determine whether individuals from a given species form a single randomly mating population or are members of different populations with various levels of genetic isolation. Indeed, genetic isolation fosters speciation opportunities (Audzijonyte, Baltrūnaitė, Väinölä, & Arbačiauskas, 2015) but may also cause extinctions of local populations (Underwood, Smith, van Oppen, & Gilmour, 2007). There is no consensus nor a general framework outlining the levels of connectivity at which populations should be considered as independent (Waples & Gaggiotti, 2006).

Genetic differentiation among populations may be observed in cases where an ancient separation is maintained with low migration rates, but also when a recent divergence arose without gene flow (Boissin, Hoareau, & Berrebi, 2011; Duda & Lessios, 2009). Indeed, geographic isolation alone is not sufficient to assess the degree of isolation (Donald, Keeney, & Spencer, 2011). Instead, population genetics provides a robust theoretical framework to estimate gene flows over multiple generations from which the degree of connectivity between pairs of populations can be assessed (Kool, Moilanen, & Treml, 2013; Wright, 1931). Traditional estimates of migration rates are based on population differentiation indices, notably Wright's *F*_*ST*_ (Wright, 1931). However, the mathematical models linking genetic variance to migration rates make numerous assumptions that are often biologically unrealistic and violated (Hedgecock et al., 2007; Whitlock & McCauley, 1999), for example, no selection nor mutation within populations. Nevertheless, indices of population differentiation can still be used with confidence in comparative studies (Meirmans & Hedrick, 2010). Additionally, individual‐based methods have been developed to highlight actual populations admixture (e.g., Jombart, Devillard, & Balloux, 2010; Pritchard, Stephens, & Donnelly, 2000), giving individual assignment probabilities to putative populations. Even though all these methods cannot estimate several population parameters (e.g., direction of migration, effective population size), identifying similar patterns of genetic structuring for multiple species with similar life history traits over the same geographic area is highly informative on the degree of population genetic connectivity, and consequently for optimizing and refining the design of marine protected areas networks.

For the majority of marine invertebrates, the adult phase is benthic with low mobility or fixed to the substrate and larvae represent the major dispersal phase ensuring population connectivity (Lopez‐Duarte et al., 2012; Selkoe & Toonen, 2011; Treml, Halpin, Urban, & Pratson, 2007) and species cohesion (Knowlton & Jackson, 1993). However, direct measures of larval dispersal are presently unfeasible as they depend on various factors that are often difficult to assess in the field, such as oceanic circulation, sea temperature, larval behavior, larval energetic resources, available habitats, and food resources [see Selkoe and Toonen (2011) for a review]. Yet this stage of the life cycle strongly influences the effective dispersal, which additionally encompasses the survival of larvae and adults, larval settlement on the substrate, and sexual reproduction with local conspecifics (Pineda, Hare, & Sponaungle, 2007). Pelagic larval duration (PLD; Shanks, 2009) is often used as a proxy for larval dispersal distance and connectivity, even though past biogeographic events affect genetic structure of marine populations (Faurby & Barber, 2012), modifying populations connectivity regardless of species PLD. Thus, the correlation between PLD and connectivity is not straightforward and must be considered cautiously (Paulay, 2006; Shanks, 2009). Indeed, if theory predicts that species presenting long PLDs will display high dispersal and thus low genetic structure, numerous examples of the contrary exist [see Weersing and Toonen (2009) for a review].

Hydrozoans represent one of the oldest marine clades, and they have colonized all aquatic ecosystems across the globe since their appearance during the Cretaceous (Bouillon, Gravili, Pagès, Gili, & Boero, 2006; Park et al., 2012). They are among the first fixed organisms to colonize new habitats and provide shelter to a wide variety of invertebrate and microbial taxa (Boero, 1984; Gili & Hughes, 1995). Despite their ecological importance and phyletic diversity, this clade is still understudied: its taxonomy is complicated and confused due to the paucity of diagnostic morphological characters, resulting in several systematic revisions and alpha‐diversity assessments during the past two decades (Bouillon & Boero, 2000; Bouillon et al., 2006; Cartwright & Nawrocki, 2010; Collins, 2002; Marques & Collins, 2004; Postaire *et al*., 2016a; Postaire *et al*., 2016b; Ronowicz et al., 2017). One of their key features is the variety of life history traits and reproductive strategies, notably including a medusa stage of variable duration depending on the taxon (Boero, Bouillon, & Piraino, 1992; Boero *et al*. 1995). The Aglaopheniidae Marktanner‐Turneretscher, 1890 is one of the most species‐rich families with more than 250 extant species found in all marine ecosystems (Millard, 1975); they are characterized by the absence of a medusa stage and by the incubation of larvae in dedicated structures, even if some species reacquired a temporally reduced medusa‐like stage during their evolution (Leclère *et al*., 2009; Leclère, Schuchert, & Manuel, 2007). Aglaopheniids’ genus‐level taxonomy is mainly based on the morphology of the reproductive structures (Bouillon et al., 2006), but as many other characters, life cycles of hydrozoans are subject to convergent or reversible evolution across their phylogeny (Collins, 2002; Leclère et al., 2007, 2009; Marques & Collins, 2004; Miglietta & Cunningham, 2012) and the diversity of Aglaopheniidae is still under assessment (Moura *et al*. 2012; Postaire et al., 2016a, 2016b).

Active dispersal in this family is thought to be limited and only achieved via spermatozoids and mature larvae (Schuchert, 2014; Winston, 2012), an assumption that was confirmed using microsatellite data for a single morpho‐species, *Lytocarpia brevirostris* (Busk, 1852), in a recent study centered on the Western Indian Ocean (Postaire *et al*., 2017). Similar results were obtained in a study of the genetic connectivity of populations on the globally invasive hydrozoan *Cordylophora* Allman, 1844, using microsatellites but with a geographically and ecologically more limited sampling centered on the North American Great Lakes basin (Darling & Folino‐Rorem, 2009). These two studies supported the idea of weak dispersal abilities in some hydrozoans due to a lack of a long dispersal phase, resulting in a pattern of high genetic differentiation among populations: One could consider each sampling site as hosting an independent biological species (Schuchert, 2014). However, more studies are needed to confirm these preliminary conclusions. Indeed, an important number of Aglaopheniidae morpho‐species, as many other hydrozoans, contradict the postulate of limited connectivity: they present global distribution ranges and occur in a wide range of habitats and depths (Millard, 1975).

One of the first steps to conduct population genetic studies is to identify the species (Pante et al., 2015). In Aglaopheniidae, integrative taxonomy (Schlick‐Steiner et al., 2010) and molecular‐based species delimitation methods allowed the delineation of robust species hypotheses in this clade (Postaire et al., 2016a). Here, the clade formed by *Macrorhynchia phoenicea* (Busk, 1852) is a typical morpho‐species presenting high morphological plasticity, asexual reproduction through stolon growth, a monophasic dioecious larviparous life cycle—that is, the larvae produced after internal fertilization are not released until competent—and an Indo‐Pacific distribution on coral reefs (Di Camillo, Puce, & Bavestrello, 2009; Millard, 1975). Species delimitation methods based on DNA revealed that this morpho‐species is actually composed of at least two sympatric cryptic species [*sensu* Bickford et al. (2007)], referred to as *M. phoenicea* morphotypes A and B in Postaire et al. (2016a) and henceforth named *M. phoenicea* α and β, respectively. They can be distinguished using a combination of general colony shape, color, microhabitats, and genetic data. The distribution ranges of both species differ, as *M. phoenicea* α is composed of two divergent lineages present in the Western Indian Ocean and the Tropical Southwestern Pacific [*sensu* Spalding et al. (2007)], whereas *M. phoenicea* β seems restricted to the Western Indian Ocean. The sexual dispersal abilities of *M. phoenicea* α are assumed to be limited in natural conditions. In laboratory conditions, larvae of *M. phoenicea* α settle in less than 24 hr (BP, pers. obs.), as found in other hydrozoan species (Sommer, 1990). Furthermore, hydrozoan sperm cells are reported to present a short planktonic life (4 hr; Yund, 1990).

To complement and confirm previous work on the population connectivity of marine hydrozoans (Darling & Folino‐Rorem, 2009; Postaire et al., 2017), intensive sampling of *M. phoenicea* α populations was conducted in the Western Indian Ocean and the Tropical Southwestern Pacific. The aims were to (i) investigate the structure and connectivity of *M. phoenicea* α populations using microsatellites (Postaire *et al*. 2015), (ii) compare the results with the study of another Aglaopheniidae with a similar reproductive strategy (Postaire et al., 2017), and (iii) discuss the distribution ranges of Aglaopheniidae species in light of our results.

## 2 MATERIALS AND METHODS

### 2.1 Sampling and DNA extraction

**Figure 1:**
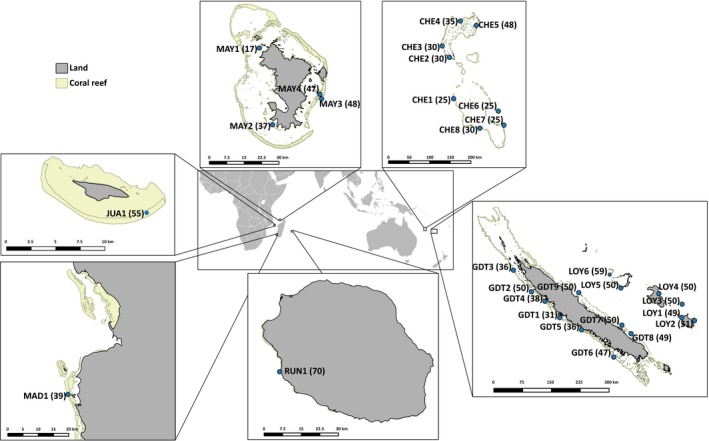
Sampling sites of *Macrorhynchia phoenicea* α in the Western Indian Ocean and the Tropical Southwestern Pacific, with site codes and the number of individuals sampled (in parentheses): CHE, Chesterfield Islands comprising Chesterfield/Bampton/Bellona Plateau; JUA, Juan de Nova Island; MAD, Madagascar; MAY, Mayotte; RUN, Reunion Island; GDT, Grande Terre; LOY, Loyalty Islands

Thirty sampling sites were explored within two marine provinces (Spalding et al., 2007): the Western Indian Ocean and the Tropical Southwestern Pacific Ocean, presenting seven islands/archipelagoes (Table 1). At each site, individuals (feather‐shaped units) were collected haphazardly using scuba during a single dive (ca. 60 min) and were placed in sequentially numbered individual bags to approximate distances between individuals; we preferentially collected individuals several centimeters apart to limit clone sampling. *Macrorhynchia phoenicea* α (see Supplementary Material 1 in Postaire et al., 2016a) was commonly found on outer reef slopes exposed to strong currents, often associated with *Pocillopora* colonies, suggesting ecological preferences in this species. Large individuals with visible reproductive structures were preferentially sampled, and all samples were stored in 95% ethanol before DNA extraction. Preliminary species identification was performed in the field (as explained in Postaire et al., 2016a) and later confirmed by detailed inspection of morphological characters (Millard, 1975) using a stereomicroscope and genotypic clustering (Postaire et al., 2016a): a total of 1,257 individuals of *M. phoenicea* α were sampled (Table 1; Figure 1). DNA was extracted from one or two ramifications using the DNeasy Blood & Tissue Kit (Qiagen, Hilden, Germany) following the manufacturer's protocol. Euclidian distances between sampling sites were measured with Google Earth v.7.1 (http://earth.google.fr/) using site coordinates (Table 1).

**Table 1:** *Macrorhynchia phoenicea* α samples (*N* = 1,257) used in this study

Marine province	Ecoregion	Archipelago/island	Site name	Site code	Latitude	Longitude	*N*	*N*_MLG_	*R*	*H*_O_	*H*_E_	*F*_*IS*_	*Ar*(11)	*Ap*(11)
WIO	Mascarene Islands	Reunion Island	Pointe au Sel	RUN1	−21.3750	55.5835	70	68	.971	0.416	0.471	0.119***	2.859 ± 0.406	0.64 ± 0.377
	West./North. Mad.	Juan de Nova Island	Biodiv 7	JUA1	−17.0747	42.7665	55	50	.907	0.264	0.437	0.359***	2.464 ± 0.278	0.31 ± 0.165
		Madagascar	Nosy Satrana	MAD1	−23.7569	43.8052	39	21	.526	0.605	0.513	−0.327***	2.360 ± 0.155	0.28 ± 0.148
		Mayotte	Récif du Boa	MAY1	−12.6857	45.0387	17	11	.625	0.420	0.506	0.176*	2.255 ± 0.244	0.05 ± 0.047
			Saziley	MAY2	−12.9826	45.1989	37	30	.861	0.446	0.610	−0.082*	2.662 ± 0.207	0.17 ± 0.075
			Passe en S	MAY3	−12.8791	45.2770	48	34	.702	0.386	0.372	−0.042^N.S.^	2.278 ± 0.285	0.05 ± 0.028
			Passe en S	MAY4	−12.8667	45.2713	47	43	.913	0.395	0.462	0.144***	2.723 ± 0.228	0.06 ± 0.023
TSP	New Caledonia	Chesterfield Islands	Ilôt Reynard	CHE1	−19.2131	158.9467	48	48	1	0.340	0.538	0.371***	3.020 ± 0.402	0.13 ± 0.079
			Récif de l'Anneau	CHE2	−19.9103	158.3618	30	30	1	0.367	0.541	0.326***	3.227 ± 0.404	0.05 ± 0.020
			Récif du Milieu	CHE3	−19.6614	158.2039	30	30	1	0.369	0.539	0.322***	3.118 ± 0.387	0.08 ± 0.076
			Récif Nord‐Ouest	CHE4	−19.1174	158.6012	35	35	1	0.332	0.549	0.411***	2.938 ± 0.406	0.09 ± 0.040
			Ilôt du Passage	CHE5	−21.3986	159.5627	25	25	1	0.391	0.556	0.302***	3.243 ± 0.339	0.16 ± 0.053
			Ilôt de Sable	CHE6	−21.0874	159.4338	25	25	1	0.370	0.529	0.305***	3.311 ± 0.399	0.09 ± 0.038
			Bampton Nord	CHE7	−21.4597	159.0315	30	28	.931	0.459	0.553	0.175***	3.297 ± 0.365	0.13 ± 0.055
			Récif Olry	CHE8	−20.8110	158.4537	25	25	1	0.361	0.526	0.318***	2.667 ± 0.365	0.05 ± 0.030
		Grande Terre	Népoui	GDT1	−21.4185	164.9671	31	31	1	0.433	0.555	0.222***	3.032 ± 0.289	0.07 ± 0.033
			Koumaé	GDT2	−20.8109	164.3055	50	45	.898	0.435	0.455	0.044^N.S.^	2.832 ± 0.345	0.04 ± 0.017
			Poum	GDT3	−20.3087	163.8854	36	31	.857	0.439	0.461	0.050^N.S.^	2.635 ± 0.282	0.04 ± 0.022
			Récif de Voh Sud	GDT4	−21.0353	164.6218	38	31	.811	0.340	0.296	−0.151**	1.870 ± 0.140	0.01 ± 0.003
			Bourail	GDT5	−21.7031	165.4757	36	33	.914	0.431	0.516	0.167***	2.787 ± 0.258	0.02 ± 0.014
			Nouméa	GDT6	−22.3406	166.2305	47	46	.978	0.398	0.424	0.060*	2.469 ± 0.233	0.05 ± 0.050
			Récif de Niaouato	GDT7	−21.5929	166.4202	50	33	.653	0.470	0.456	−0.030^N.S.^	2.340 ± 0.243	0.02 ± 0.015
			Récif du Solitaire	GDT8	−21.7918	166.6352	49	43	.875	0.307	0.422	0.274***	2.613 ± 0.271	0.06 ± 0.035
			Ile Sable	GDT9	−20.8346	165.4124	50	23	.449	0.359	0.287	−0.257***	1.729 ± 0.152	0.09 ± 0.046
		Loyalty Islands	Maré; Sud Cap Machan	LOY1	−21.4161	167.8192	49	44	.896	0.418	0.491	0.151***	2.283 ± 0.234	0.02 ± 0.017
			Maré; Sud de Cap Coster	LOY2	−21.4867	168.1194	51	46	.900	0.368	0.460	0.202***	2.316 ± 0.241	0.02 ± 0.017
			Tiga	LOY3	−21.1032	167.8285	50	49	.980	0.335	0.376	0.109***	2.294 ± 0.279	0.01 ± 0.005
			Lifou	LOY4	−20.8554	167.2860	50	50	1	0.352	0.385	0.083*	2.293 ± 0.253	0.04 ± 0.024
			Ouvea	LOY5	−20.7244	166.3965	50	36	.714	0.361	0.377	0.038^N.S.^	2.337 ± 0.254	0.02 ± 0.010
			Beautemps Beaupré	LOY6	−20.4144	166.1384	59	37	.621	0.401	0.364	−0.102*	2.012 ± 0.160	0.05 ± 0.029

For each sampling site, the following are indicated: GPS coordinates (in decimal degrees), total sample size (*N*), number of multilocus genotypes (*N*_MLG_), clonal richness *R* = [(*N*_MLG_ − 1)/(*N *− 1)], observed (*H*_O_) and expected (*H*_E_) heterozygosities, inbreeding coefficient (*F*_IS_), allelic richness *Ar*(11) ± SE (standard error), and private allelic richness *Ap*(11) ± SE. *H*_O_, *H*_E_, and *F*_IS_ were calculated keeping one representative per MLG and per sampling site. With the *F*_IS_ is indicated the test significance for deviation to Hardy–Weinberg equilibrium: N.S.: nonsignificant; *p < 0.05; **p < 0.01; ***p < 0.001.

### 2.2 Microsatellite genotyping

We used 26 microsatellite loci specific to the morpho‐species *M. phoenicea* (Postaire et al., 2015), using the same PCR conditions. PCR products were genotyped using an ABI 3730 genetic analyzer (Applied Biosystems), and allelic sizes were determined on GeneMapper v.4.0 (Applied Biosystems) using an internal size standard (GeneScan LIZ‐500; Applied Biosystems). Considering the whole dataset, over the 26 available loci for *M. phoenicea* sp., 15 amplified correctly *M. phoenicea* α individuals, that is, presented less than 10% of missing data, and were considered for all analyses. For each sampling site, identical multilocus genotypes (MLGs) were identified with GenClone v.2.0 and clonal richness *R* [(*N*_MLG_ − 1)/(*N* − 1)] was assessed (Arnaud‐Haond & Belkhir, 2007; Table 1).

### 2.3 Summary statistics

One representative of each MLG per site was used for further analyses. All tests in this study were corrected for false discovery rate (FDR) in multiple tests (Benjamini & Hochberg, 1995). We used Micro‐Checker v.2.3 (van Oosterhout *et al*., 2004) to check for scoring errors and to estimate null allele frequencies. Linkage disequilibrium (LD) was tested using Arlequin v.3.5 (Excoffier & Lischer, 2010) among all pairs of loci within each site with 10^3^ permutations. Observed (*H*_*O*_) and expected (*H*_E_) heterozygosities and tests for Hardy–Weinberg equilibrium (HWE) were computed using the software Arlequin v.3.5 (Excoffier & Lischer, 2010) within all sites and over all loci. Average allelic richness and private allelic richness were compared among each site using HP‐RARE (Kalinowski, 2005) software to correct for uneven sample sizes by rarefaction. The software sampled 11 individuals at random from each site to match the smallest sample size (i.e., MAY1; Table 1).

### 2.4 Population differentiation

We investigated population differentiation and structure using four different approaches: pairwise comparisons among sites, discriminant analysis of principal components (individual level), Bayesian clustering (individual level), and network construction (site and individual levels). First, the geographic origin of individuals (i.e., site) was treated as an a priori defined population, except in clustering analyses. Pairwise *F*_*ST*_ (Wright, 1931) comparisons among sites was conducted with Arlequin v.3.5 (Excoffier & Lischer, 2010); the significance of the observed *F*_*ST*_‐statistics was tested using the null distribution generated from 5 × 10^3^ nonparametric random permutations. Jost's *D* (Jost, 2008) comparisons among sites were conducted with GENODIVE v.2.0 (Meirmans & van Tienderen, 2004); the significance of the observed Jost's *D*‐statistics was tested with DEMEtics (Gerlach *et al*. 2010), which uses a bootstrap method (1,000 bootstrap repeats) to estimate *p*‐values. Fisher's exact tests of site differentiation based on genic frequencies (Raymond & Rousset, 1995a>) were performed in Genepop v.4.6 (Raymond & Rousset, 1995b). To understand the mechanisms that may be responsible for the observed patterns of population structure, we compared estimates of genetic differentiation to geographic distances among sites. We used a Mantel test (Mantel, 1967) to evaluate the correlation between linearized genetic differentiation [Slatkin's distance: (*F*_*ST*_/(1 − *F*_*ST*_)] and the straight‐line geographic distance [ln(distance)] among sites (Table 2). This relationship is expected to be positive and linear in the context of a two‐dimensional Isolation by distance (IBD) model (Rousset, 1997). All Mantel tests were performed using the program GENODIVE v.2.0 (Meirmans & van Tienderen, 2004) with 10^4^ random permutations to assess significance.

**Table 2:** *Macrorhynchia phoenicea* α pairwise *F*_*ST*_ values among sampling sites from the Western Indian Ocean and the Tropical Southwestern Pacific

Site	RUN1	JUA1	MAD1	MAY1	MAY2	MAY3	MAY4
CHE1	0.490	0.563	0.495	0.516	0.493	0.580	0.493
CHE2	0.486	0.534	0.481	0.511	0.490	0.567	0.488
CHE3	0.472	0.528	0.482	0.484	0.470	0.547	0.462
CHE4	0.460	0.536	0.475	0.484	0.478	0.557	0.470
CHE5	0.461	0.523	0.466	0.460	0.453	0.520	0.451
CHE6	0.470	0.520	0.461	0.460	0.438	0.526	0.441
CHE7	0.466	0.528	0.471	0.473	0.458	0.545	0.460
CHE8	0.512	0.579	0.526	0.542	0.517	0.595	0.512
GDT1	0.433	0.484	0.430	0.451	0.447	0.521	0.445
GDT2	0.478	0.528	0.491	0.494	0.474	0.539	0.471
GDT3	0.497	0.519	0.480	0.523	0.506	0.573	0.492
GDT4	0.499	0.584	0.570	0.589	0.565	0.618	0.530
GDT5	0.453	0.506	0.453	0.463	0.455	0.521	0.446
GDT6	0.467	0.519	0.488	0.497	0.477	0.536	0.466
GDT7	0.514	0.543	0.513	0.525	0.505	0.564	0.481
GDT8	0.500	0.515	0.494	0.497	0.482	0.533	0.457
GDT9	0.602	0.640	0.638	0.645	0.556	0.626	0.532
LOY1	0.519	0.538	0.515	0.528	0.503	0.567	0.490
LOY2	0.507	0.524	0.509	0.515	0.490	0.558	0.473
LOY3	0.543	0.580	0.549	0.566	0.529	0.589	0.521
LOY4	0.524	0.549	0.530	0.531	0.507	0.562	0.480
LOY5	0.550	0.540	0.547	0.539	0.508	0.567	0.479
LOY6	0.554	0.558	0.576	0.556	0.525	0.582	0.495
Mean	0.498	0.541	0.506	0.515	0.492	0.561	0.480
Standard error	0.008	0.007	0.010	0.010	0.007	0.006	0.005

All *F*_*ST*_ values were highly significantly different from 0 (*p* < 0.001) after FDR correction.

### 2.5 Clustering analyses

Population structuring was also assessed without a priori stratification of samples. We first performed a discriminant analysis of principal components (DAPC) using the package *adegenet* (Jombart, 2008; Jombart et al., 2010) in R v.3.2.3 (R Development Core Team 2004). DAPC is a non‐model‐based method that maximizes the differences among groups while minimizing variation within groups without prior information on individuals’ origin. In addition, the method does not assume HWE or absence of LD. We used the function find.clusters() to assess the optimal number of groups with the Bayesian information criterion (BIC) method (i.e., K with the lowest BIC value is ideally the optimal number of clusters). Note that BIC values may keep decreasing after the true K value in case of genetic clines and hierarchical structure (Jombart et al., 2010) and that retaining too many discriminant functions with respect to the number of populations may lead to overfitting the discriminant functions, resulting in spurious discrimination of any set of clusters. Therefore, the rate of decrease in BIC values was visually examined to identify values of K after which BIC values decreased only subtly (Jombart et al., 2010); we tested values of K = 1–30. The dapc() function was then executed using the best grouping, retaining axes of PCA sufficient to explain ≥70% of total variance of data, and coloring individuals according to their sampling site.

**Figure 2:**
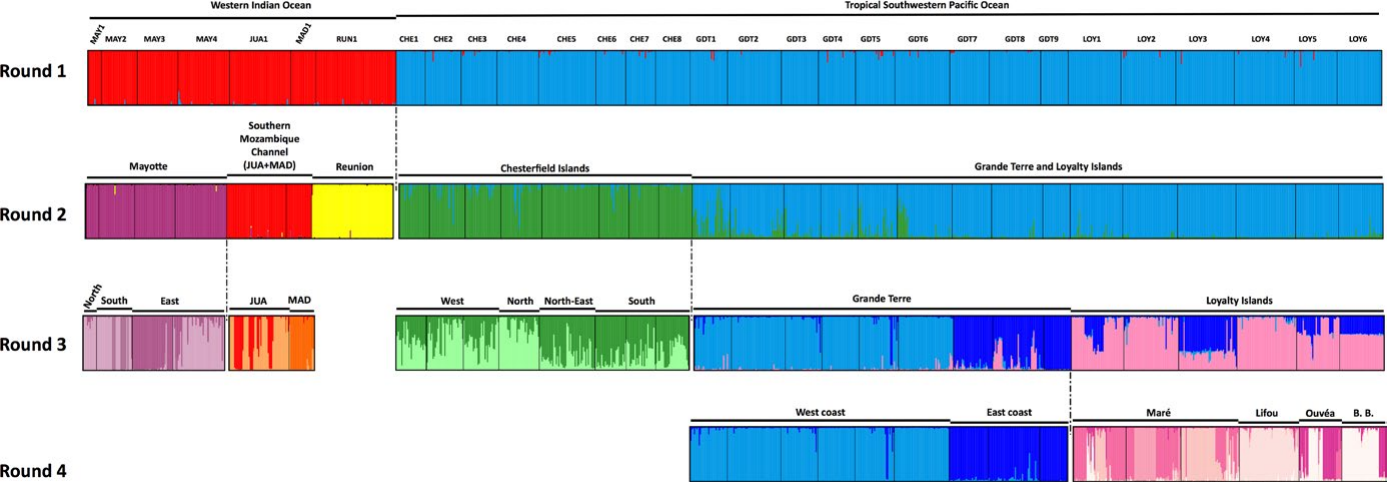
*Macrorhynchia phoenicea* α. Assignment probabilities of multilocus genotypes (MLGs) to putative clusters using an admixture model as identified by Structure. Round 1: average probability of membership (*y*‐axis) of MLGs (*N*_MLG_ = 1,081, *x*‐axis) in *K* = 2 clusters. Round 2: average probability of membership (*y*‐axis) of MLGs from the Western Indian Ocean (left, *N*_MLG_ = 257, *x*‐axis) in *K* = 3 clusters and the Tropical Southwestern Pacific (right, *N*_MLG_ = 824, *x*‐axis) in *K* = 2. Round 3: average probability of membership (*y*‐axis) of MLGs from the Chesterfield Islands sites (left, *N*_MLG_ = 246, *x*‐axis) in *K* = 2 clusters and the Grande Terre/the Loyalty Islands sites (right, *N*_MLG_ = 578, *x*‐axis) in *K* = 3. Round 4: average probability of membership (*y*‐axis) of MLGs from the Grande Terre sites (left, *N*_MLG_ = 316, *x*‐axis) in *K* = 2 clusters and the Loyalty Islands sites (right, *N*_MLG_ = 262, *x*‐axis) in *K* = 6 . B.B.: Beautemps Beaupré.

The population clustering was also explored using the software Structure v.2.3.2 (Pritchard, Wen, & Falush, 2010; Pritchard et al., 2000), with the admixture model and correlated allele frequencies (Falush & Pritchard, 2003). This analysis assumes that within the analyzed dataset reside *K* populations, and individuals are assigned probabilistically to each population in order to maximize HWE and minimize LD. Due to the important size of our dataset and following the recommendations of Rosenberg et al. (2002) and Jakobsson et al. (2008), we studied our dataset using a hierarchical approach. For each group of sites (Figure 2) and each tested value of *K* (*K* varying from 1 to 10), three independent runs were conducted with a burn‐in period of 5 × 10^4^ steps followed by 5 × 10^5^ Markov chain Monte Carlo iterations. We used the statistic proposed by Evanno, Regnaut, and Goudet (2005), implemented in Structure Harvester v.1.0 (Earl & vonHoldt, 2012), to estimate the best number of *K* for each group of sites. The software CLUMPP v.1.0 (Jakobsson & Rosenberg, 2007) was used to summarize results, and they were formatted with DISTRUCT v.1.1 (Rosenberg, 2004). The software Arlequin v.3.5 (Excoffier & Lischer, 2010) was then used to perform hierarchical analyses of molecular variance using clusters identified by Structure as populations, which mostly corresponded to islands/archipelagoes, and provinces as groups.

Finally, network analyses were performed on individuals and sites. The pattern of genetic relationship among individuals was illustrated by networks built with two measures integrating genetic information in terms of time and divergence history: the Rozenfeld Distance index (RD) and the Shared Allele Distance index (SAD). RD has been developed from the Goldstein distance index. It provides a parsimonious representation of the genetic distance between individuals based on the difference of the microsatellites allele lengths (Rozenfeld et al., 2007). On the other hand, SAD provides the genetic distance between individuals based on the proportion of shared alleles (Chakraborty & Jin, 1993). RD helps to resolve ancestral polymorphism through allele lengths impinged on slow evolutionary processes, while SAD helps to understand recent gene flow characterized by direct allelic exchange.

The global pattern of genetic relationships among sites was illustrated by networks built with two different measures: the Goldstein distance index (GD) and *F*_*ST*_ fixation index (*F*_*ST*_). The GD groups sites considering their historical origin, while *F*_*ST*_ takes into account the site structure. Once the matrices of genetic distances between individuals or sites were estimated, different networks were built considering individuals/sites and genetic distances as nodes and links between them, respectively. For the network construction, links were included for all distances and were removed in decreasing order until the percolation threshold (Dpe) was reached (Rozenfeld et al., 2007), threshold below which the network fragmented into small clusters. The average clustering coefficient < C > of the whole network was estimated for each of the four built networks. These analyses were performed using EDENetworks software (Kivelä, Arnaud‐Haond, & Saramäki, 2015).

## 3 RESULTS

### 3.1 Multilocus genotyping and asexual reproduction

Using the 15 loci that amplified correctly (see Section 2.2), our analysis of 1,257 individuals yielded 1,081 MLGs, indicating the presence of asexual reproduction in some sites (Table 1). Individuals sharing the same MLG were always found within the same site (i.e., no MLGs were shared among sites) and were found close to one another (i.e., small difference in sampling numbers). The clonal richness *R* ranged from 0.449 to 1.

### 3.2 Genetic variability

All loci were polymorphic, with a total number of alleles ranging from five (Mp20) to 27 (Mp24) [mean ± standard error (*SE*) = 14.8 ± 1.7], with some loci monomorphic in several sites (Table S1). Significant LD among loci was detected in the complete dataset (*p* < .05, 1,421 tests over 3,150 after FDR correction, i.e., 45.11%). However, nearly 50% of the positive tests (700 of 1,421) occurred due to monoallelic loci in various sites and might just reflect their general low genetic diversity. Observed heterozygosities ranged from 0.264 to 0.605 (mean ± *SE* = 0.392 ± 0.011) in JUA1 and MAD1, respectively, and unbiased expected heterozygosities from 0.287 to 0.610 (mean ± *SE* = 0.468 ± 0.015) in GDT9 and MAY2, respectively. Mean allelic richness per locus ranged from 1.729 ± 0.151 in GDT9 to 3.311 ± 0.399 in CHE6 and mean number of private allele per locus ranged from 0.005 ± 0.003 in GDT4 to 0.314 ± 0.165 in JUA1. Multilocus *F*_*IS*_ values ranged from −0.327*** for MAD1 to 0.411*** for CHE4 (Table 1). Null alleles were detected for several loci in multiple sites. However, as (i) LD was inconstant among loci, (ii) not a single locus was monomorphic over all sites, (iii) the number of null alleles was inconstant among sites, and (iv) the value of *F*_*IS*_ was found significantly positive or negative whatever the presence or absence of null alleles, we decided to keep the 15 loci for further analyses.

### 3.3 Genetic clusters

Both DAPC and Structure analyses indicated significant structuring of sites, with MLGs clustering according to their geographic origin. The first round of Structure analyses identified two clusters, each corresponding to one province (Figure 2, Round 1); both provinces were subsequently analyzed separately. In the Western Indian Ocean, Structure identified three clusters: one corresponding to Reunion Island (RUN1) and the other two corresponding to the northern (MAY1 to MAY5) and southern (JUA1 and MAD1) parts of the Western and Northern Madagascar ecoregion (Spalding et al., 2007). In the Tropical Southwestern Pacific, two clusters were identified, corresponding either to the western part (the Chesterfield Islands) or eastern part (Grande Terre and the Loyalty Islands) of the New Caledonia ecoregion (Spalding et al., 2007; Figure 2, Round 2). Once again, both clusters were analyzed separately. The MLGs from the Chesterfield Islands were assigned to two clusters that seemed to correspond to geography but with high admixture: West and North versus East and South. For the MLGs from Grande Terre and the Loyalty Islands, the clustering was not entirely stable, but a first consensus emerged at *K* = 3 with two clusters corresponding to Grande Terre (GDT1 to GDT9), whereas MLGs from the Loyalty Islands (LOY1 to LOY6) were poorly assigned to two clusters (Figure 2, Round 3). As the clustering scheme for MLGs from the Loyalty Islands was variable among runs, contrary to MLGs from Grande Terre that seemed strongly assigned to their clusters, we decided to analyze Grande Terre and the Loyalty Islands separately. In this fourth round, the MLGs from Grande Terre clustered according to their origin, that is, West (GDT1 to GDT6) and East (GDT7 to GDT9) coasts, as did MLGs from the Loyalty Islands, but with a relatively important number of clusters (*K* = 6) and some admixture (Figure 2, Round 4).

**Figure 3:**
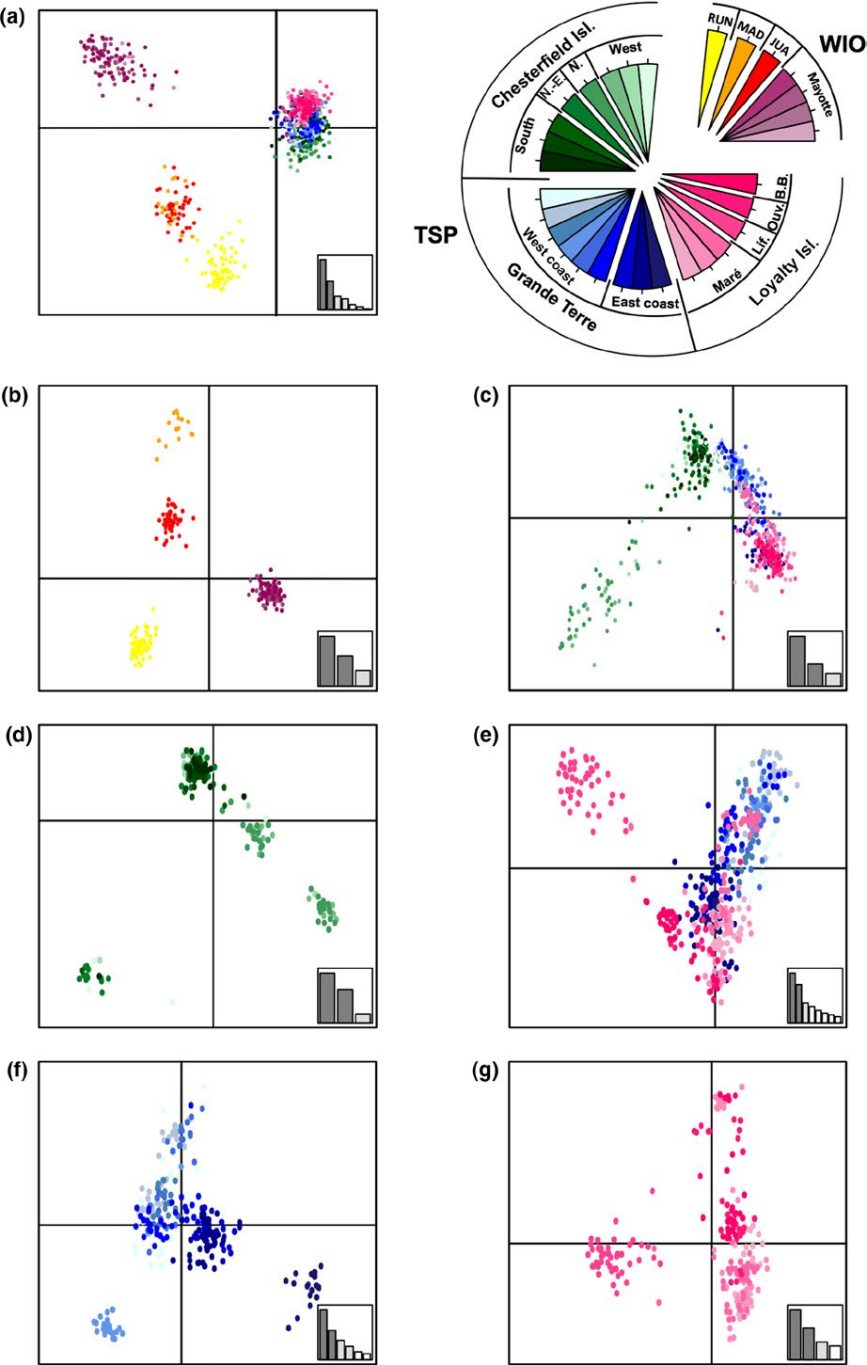
*Macrorhynchia phoenicea* α. Discriminant analysis of principal components (DAPC) of multilocus genotypes (MLGs) sampled in the Western Indian Ocean (WIO) and the Tropical Southwestern Pacific (TSP) ecoregions. Scatter plots of the MLGs from (a) both ecoregions, (b) the Western Indian Ocean, (c) the Tropical Southwestern Pacific, (d) the Chesterfield Islands, (e) Grande Terre and the Loyalty Islands, (f) Grande Terre, and (g) the Loyalty Islands using the first and second components. MLGs are colored according to their geographic origin (island/archipelago). RUN, Reunion Island; MAD, Madagascar; JUA, Juan de Nova Island; Lif.: Lifou; Ouv.: Ouvea; B.B.: Beautemps Beaupré.

DAPC results were concordant with Structure outputs and we used the same hierarchical approach. When analyzing the whole dataset, even if there was no clear value of K as BIC decreased steadily until reaching K = 30 (data not shown), DAPC identified a clear distinction between MLGs from Western Indian Ocean and the Tropical Southwestern Pacific (Figure 3a). When analyzing only MLGs from the Western Indian Ocean, they clustered according to their origin (i.e., island; Figure 3b). MLGs from the Tropical Southwestern Pacific clustered according to their origin with some admixture as in Structure (Figure 3c), but also without a clear value of K. During data analysis, we noted that the presence of missing data in the loci Mp08 and Mp15 induced the formation of two symmetrical groups of clusters with the same previous geographic signal within the Tropical Southwestern Pacific (data not shown). As the two groups of clusters were constant in all subsequent partitioning of the dataset, and in order to ease the results interpretation, we decided to prune from the dataset all MLGs presenting missing data for both loci (only for DAPCs). Among MLGs from the Chesterfield Islands, DAPC identified more clusters than Structure, but without geographic grouping (Figure 3d), contrary to the MLGs from Grande Terre and the Loyalty Islands, which clustered according to their origin with some admixture (Figure 3e). The clustering of MLGs from Grande Terre was similar to Structure, with a split between western and eastern sites (Figure 3f). The clustering scheme of the Loyalty Islands was also similar to Structure, with a fuzzy geographic signal (North vs. South) and with MLGs from Lifou clustering separately from the others (Figure 3g).

### 3.4 Network analysis

**Figure 4:**
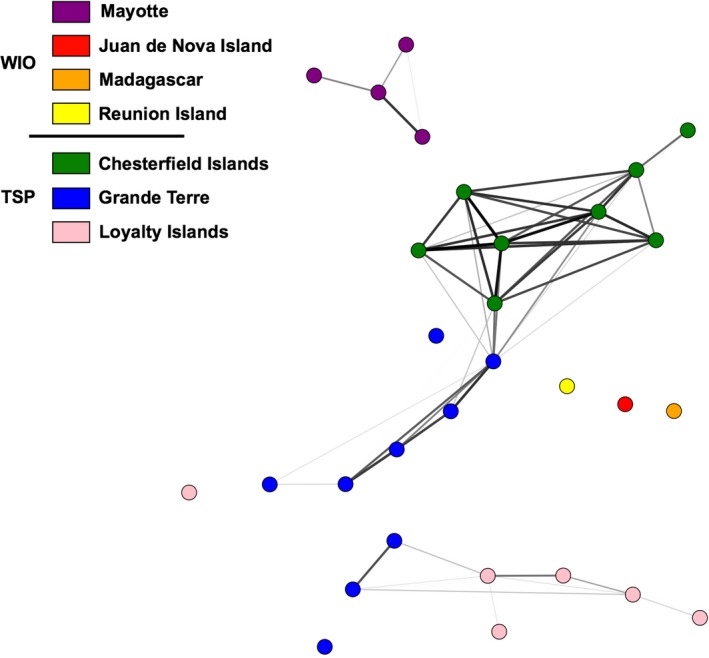
*Macrorhynchia phoenicea* α. Network topology of the 30 sampling sites, based on pairwise *F*_*ST*_ values. Only links with distances smaller than or equal to the percolation threshold (Dpe = 0.18) are presented. Nodes representing sampling sites are colored according to their geographic origin (island/archipelago). WIO, Western Indian Ocean; TSP, Tropical Southwestern Pacific

The topology of the network built with the SAD index at the percolation threshold (Dpe = 0.92) showed that individuals of one island/archipelago remained linked and were more closely related to each other than to individuals from other islands/archipelagoes. In contrast, the network built with the RD index (Dpe = 5.62) resulted in less geographic structure among MLGs (Fig. S1). The average clustering coefficient was lower for the network built with the SAD index (< C > = 0.68) than for the network built with the RD index (< C > = 0.73). The topology of the network based on pairwise *F*_*ST*_ values (< C > = 0.54) at the percolation threshold (Dpe = 0.18) revealed strong relationships among sites from the same island/archipelago, especially in the New Caledonia ecoregion (Figure 4), while some islands appeared not connected (Reunion Island, Juan De Nova Island, Madagascar). Moreover, the network built with the GD index (<C> = 0.90) at the percolation threshold (Dpe = 56.29) indicated two clear groups corresponding to the Western Indian Ocean and the Tropical Southwestern Pacific provinces (Fig. S1).

Using provinces as groups and islands/archipelagoes as populations, AMOVA revealed highly significant genetic structuring at all levels: between provinces (the Western Indian Ocean and the Tropical Southwestern Pacific), among islands within provinces, and within islands (Table S1). The genetic variation explained by differences between provinces was higher than the genetic variation explained by differences among islands within provinces (23.51%* and 17.52%***, respectively), but the highest amount of genetic variation was found among sites within islands (58.97%***).

### 3.5 Population differentiation and isolation by distance

All pairwise *F*_*ST*_ and Jost's *D* differentiation tests, as well as exact Fisher's tests, were significant (after FDR correction). Pairwise *F*_*ST*_ values indicated high differentiation among all sites, ranging from 0.035*** to 0.645*** (mean ± *SE* = 0.348 ± 0.008), as did Jost's *D* values (Table S2). On average, the higher *F*_*ST*_ values occurred when comparing sites between both provinces, ranging from 0.430*** between MAD1 and GDT1 and 0.645*** between MAY1 and GDT9 (mean ± *SE* = 0.513 ± 0.004; Table 2). Concerning the sites from the Western Indian Ocean, *F*_*ST*_ values ranged from 0.063*** between MAY2 and MAY4 to 0.581*** between JUA1 and MAY3 (mean ± *SE* = 0.384 ± 0.038; Table 3). In the Tropical Southwestern Pacific, *F*_*ST*_ values ranged from 0.035*** between CHE2 and CHE4 to 0.558*** between GDT4 and GDT9 (mean ± *SE* = 0.240 ± 0.007; Table 4). The lowest differentiation values recorded in our sampling were measured among sites from the Chesterfield Islands (mean ± *SE* = 0.064 ± 0.004), with a maximum of 0.111*** between CHE3 and CHE8. Overall, the differentiation among sites from Grande Terre or among those from the Loyalty Islands was approximately half that of the differentiation that existed among the Western Indian Ocean sites: Within Grande Terre, values ranged from 0.064*** between GDT1 and GDT2 to 0.558*** between GDT4 and GDT9 (mean ± *SE* = 0.225 ± 0.020); and within the Loyalty Islands, from 0.095*** between LOY1 and LOY2 to 0.340*** between LOY3 and LOY6 (mean ± *SE* = 0.227 ± 0.020).

**Table 3:** *Macrorhynchia phoenicea* α pairwise *F*_*ST*_ (below diagonal) and Jost's *D* (above diagonal) values among sampling sites from the Western Indian Ocean

Site	RUN1	JUA1	MAD1	MAY1	MAY2	MAY3	MAY4
RUN1		0.566	0.685	0.674	0.710	0.762	0.714
JUA1	0.449		0.320	0.767	0.754	0.776	0.681
MAD1	0.460	0.326		0.800	0.720	0.744	0.701
MAY1	0.466	0.558	0.516		0.133	0.164	0.095
MAY2	0.459	0.521	0.459	0.143		0.129	0.059
MAY3	0.524	0.581	0.544	0.219	0.157		0.079
MAY4	0.462	0.493	0.457	0.105	0.063	0.101	

All *F*_*ST*_ and Jost's *D* values were highly significantly different from 0 (*p* < 0.001) after FDR correction.

**Table 4:** *Macrorhynchia phoenicea* α pairwise *F*_*ST*_ (below diagonal) and Jost's *D* (above diagonal) values among sampling sites from the Tropical Southwestern Pacific

Site	CHE1	CHE2	CHE3	CHE4	CHE5	CHE6	CHE7	CHE8	GDT1	GDT2	GDT3	GDT4	GDT5	GDT6	GDT7	GDT8	GDT9	LOY1	LOY2	LOY3	LOY4	LOY5	LOY6
CHE1		0.050	0.081	0.077	0.066	0.063	0.048	0.047	0.152	0.211	0.284	0.347	0.282	0.285	0.322	0.386	0.451	0.313	0.364	0.317	0.477	0.393	0.323
CHE2	0.055		0.045	0.030	0.063	0.059	0.036	0.054	0.113	0.168	0.229	0.257	0.239	0.222	0.240	0.308	0.370	0.258	0.331	0.253	0.399	0.359	0.320
CHE3	0.086	0.047		0.053	0.054	0.068	0.051	0.099	0.126	0.155	0.199	0.232	0.212	0.187	0.224	0.299	0.406	0.262	0.317	0.292	0.327	0.306	0.257
CHE4	0.085	0.035	0.059		0.080	0.109	0.073	0.094	0.115	0.169	0.214	0.236	0.233	0.258	0.249	0.319	0.396	0.304	0.370	0.265	0.404	0.382	0.347
CHE5	0.065	0.062	0.054	0.081		0.066	0.046	0.071	0.110	0.145	0.211	0.253	0.198	0.237	0.243	0.303	0.389	0.260	0.315	0.265	0.392	0.328	0.306
CHE6	0.061	0.056	0.064	0.102	0.059		0.042	0.059	0.149	0.207	0.295	0.338	0.278	0.267	0.257	0.320	0.388	0.247	0.314	0.284	0.379	0.309	0.251
CHE7	0.050	0.037	0.052	0.077	0.044	0.039		0.031	0.107	0.177	0.243	0.278	0.244	0.238	0.262	0.320	0.399	0.250	0.313	0.283	0.401	0.332	0.269
CHE8	0.058	0.064	0.111	0.108	0.075	0.064	0.036		0.134	0.197	0.292	0.319	0.286	0.298	0.297	0.346	0.408	0.283	0.354	0.280	0.443	0.369	0.297
GDT1	0.136	0.103	0.115	0.110	0.095	0.120	0.094	0.132		0.063	0.100	0.175	0.088	0.147	0.182	0.214	0.366	0.213	0.224	0.177	0.307	0.269	0.267
GDT2	0.203	0.165	0.155	0.172	0.135	0.181	0.165	0.204	0.064		0.063	0.208	0.094	0.183	0.182	0.214	0.369	0.219	0.218	0.171	0.301	0.241	0.274
GDT3	0.266	0.219	0.198	0.216	0.190	0.246	0.220	0.287	0.099	0.072		0.219	0.061	0.184	0.152	0.171	0.377	0.198	0.177	0.183	0.287	0.222	0.265
GDT4	0.375	0.295	0.278	0.287	0.263	0.338	0.305	0.372	0.204	0.250	0.277		0.198	0.199	0.226	0.274	0.456	0.368	0.361	0.280	0.296	0.385	0.371
GDT5	0.242	0.209	0.191	0.212	0.167	0.217	0.203	0.261	0.081	0.097	0.068	0.236		0.135	0.147	0.162	0.352	0.184	0.181	0.145	0.263	0.231	0.262
GDT6	0.270	0.218	0.190	0.247	0.214	0.235	0.223	0.294	0.145	0.189	0.198	0.255	0.142		0.209	0.231	0.411	0.222	0.231	0.218	0.182	0.247	0.247
GDT7	0.306	0.240	0.229	0.255	0.224	0.237	0.249	0.306	0.178	0.195	0.177	0.298	0.158	0.23		0.058	0.182	0.098	0.133	0.131	0.235	0.128	0.202
GDT8	0.332	0.278	0.273	0.291	0.258	0.269	0.278	0.325	0.196	0.214	0.186	0.321	0.164	0.239	0.076		0.188	0.117	0.127	0.158	0.242	0.098	0.161
GDT9	0.466	0.398	0.423	0.418	0.372	0.392	0.409	0.459	0.367	0.389	0.422	0.558	0.374	0.435	0.276	0.26		0.240	0.282	0.265	0.394	0.259	0.331
LOY1	0.298	0.253	0.256	0.287	0.238	0.230	0.241	0.293	0.204	0.226	0.218	0.401	0.190	0.241	0.128	0.143	0.322		0.071	0.102	0.226	0.106	0.189
LOY2	0.332	0.304	0.295	0.333	0.276	0.277	0.284	0.342	0.212	0.225	0.200	0.396	0.188	0.248	0.167	0.154	0.359	0.095		0.185	0.235	0.083	0.189
LOY3	0.330	0.274	0.304	0.288	0.262	0.283	0.290	0.318	0.194	0.203	0.225	0.363	0.173	0.257	0.181	0.200	0.374	0.145	0.234		0.224	0.200	0.261
LOY4	0.423	0.371	0.326	0.379	0.341	0.341	0.362	0.421	0.291	0.306	0.310	0.374	0.272	0.222	0.282	0.275	0.467	0.270	0.278	0.289		0.209	0.218
LOY5	0.376	0.344	0.309	0.363	0.300	0.294	0.318	0.379	0.262	0.260	0.259	0.445	0.246	0.279	0.176	0.133	0.377	0.148	0.120	0.269	0.275		0.083
LOY6	0.353	0.337	0.292	0.363	0.300	0.270	0.292	0.348	0.276	0.300	0.310	0.456	0.285	0.293	0.269	0.213	0.459	0.249	0.250	0.340	0.298	0.143	

All *F*_ST_ and Jost's *D* values were highly significantly different from 0 (*p* < 0.001) after FDR correction.

**Figure 5:**
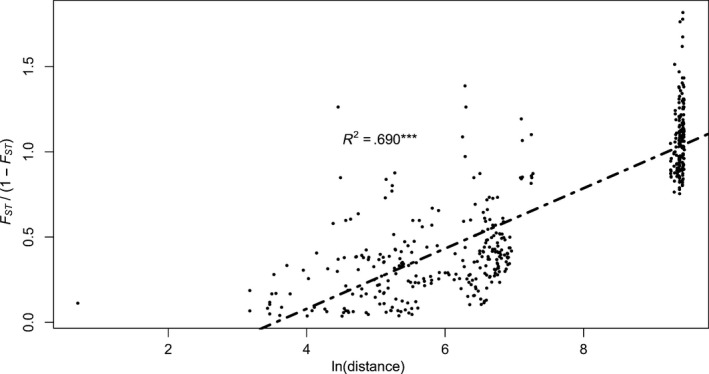
Mac*rorhynchia phoenicea* α. Correlation between genetic distances computed as *F*_*ST*_/(1 − *F*_*ST*_) and the ln of geographic distances (in kilometers) between pairs of sites at the Indo‐Pacific scale

Mantel tests revealed a significant positive correlation between transformed *F*_*ST*_ values and the ln of the geographic distances among sites (*n* = 30, *r* = 0.831***, *R* ^2^ = 0.690; Figure 5), suggesting a strong IBD pattern. This pattern was also present both within the Western Indian Ocean (*n* = 7, *r* = 0.819*, *R* ^2^ = 0.671; Fig. S1) and, in the lesser extent, the Tropical Southwestern Pacific (*n* = 23, *r* = 0.314**, *R* ^2^ = 0.098; Fig. S2). However, at lower geographic scales, IBD was detected only in the Chesterfield Islands (*n* = 8, *r* = 0.388*, *R* ^2^ = 0.150), but not within islands of the Western Indian Ocean (i.e., among sites from Mayotte) or in New Caledonia (sites from Grande Terre and the Loyalty Islands, either together or independently).

## 4 DISCUSSION

We explored the population genetic structuring and connectivity of a widely distributed hydrozoan, *Macrorhynchia phoenicea* α (Postaire et al., 2016a) across multiple geographic scales in the Indian and the Pacific Oceans using 15 newly developed microsatellite loci. This is one of the first and most extensive studies on a marine brooding hydrozoan to date, both in terms of sampling size and geographic extent, and encompassing two understudied marine regions: the Western Indian Ocean and the Tropical Southwestern Pacific (New Caledonia and associated islands). Our results revealed a high level of genetic differentiation among sites across the Indo‐Pacific at all spatial scales, with strong isolation by distance, and with genetic clusters mostly corresponding to islands. Our findings are in accordance with a growing body of literature highlighting the extreme spatial structuring of marine hydrozoans that lack a medusa dispersal stage (Postaire et al., 2017; Schuchert, 2005, 2014).

### 4.1 Life history traits affect genetic diversity

*Macrorhynchia phoenicea* α showed departures from HWE in almost all sites, generally with significant heterozygote deficit (revealed by high positive *F*_*IS*_ values). However, this result could be explained by the presence of null alleles which may occur due to some mutations in the flanking regions of microsatellite loci (Callen et al., 1993). Yet, while these are important to reveal, null alleles have little effect on structuring analyses when populations are strongly differentiated (Carlsson, 2008; Putman & Carbone, 2014), as observed here. Biological processes, such as nonrandom mating between individuals, inbreeding and/or Wahlund effects, probably also contribute to the heterozygote deficit within sites. Dioecious Aglaopheniidae species, like *M. phoenicea* α, are generally larviparous and several life history traits (supposed limited larval dispersal abilities and reproduction between spatially proximate individuals) intuitively enhance self‐recruitment and minimize emigration out of settled populations: larvae that settle quickly should remain close to the mother individual if they encounter suitable environmental conditions, thus forming patches of related individuals over several generations. This assumption, however, has not been tested yet.

Heterozygosity deficiencies could also be due to a temporal Wahlund effect resulting from (i) different cohorts at each site or (ii) different breeding units among sampling sites, as proposed to explain the high heterozygosity deficiencies in Caribbean sponges (Chaves‐Fonnegra, 2014; Duran, Pascual, & Turon, 2004). Indeed, the availability of food and oxygen are the main limiting resources for growth, sexual reproduction, and gamete production in hydrozoans (reviewed in Gili & Hughes, 1995). Thus, local conditions (water flow, temperature, planktonic productivity, sedimentation) could result in desynchronized reproduction among individuals in the population, favoring inbreeding. This is a plausible hypothesis as *M. phoenicea* α appears to reproduce throughout the year (BP pers. obs.), similarly to the tropical aglaopheniid hydrozoan *Lytocarpia brevirostris* (Postaire et al., 2017). Inbreeding might also be fostered by a spatial Wahlund effect: the sampling of a site might comprise several spatially distinct subpopulations. Indeed, the low density of *M. phoenicea* α at some sampling sites necessitated increasing the sampling area in order to collect an adequate number of individuals, thus possibly resulting in sampling different subpopulations. Basic ecological data (e.g. life span of a genet, number of reproductive events, growth rates, sex ratio) and cohort studies are necessary to resolve these issues, but this is particularly difficult in hydrozoans due to their relative small size and numerous hidden stages (Gili & Hughes, 1995). Conversely, one could argue that several populations of *M. phoenicea* α presented an excess of heterozygosity, contradicting the general pattern of kinship reproduction. However, this excess can be related to another life history trait of *M. phoenicea* α: in small populations of clonal organisms, such as Aglaopheniidae (Bouillon et al., 2006; Gili & Hughes, 1995), long periods of asexual reproduction can lead to negative *F*_*IS*_ values (Balloux, Lehmann, & de Meeus, 2003; Stoeckel & Masson, 2014). Thus, life history traits seem to profoundly affect genetic diversity at the site scale (<200 m).

### 4.2 Small‐scale spatial genetic structure and diversity

*Macrorhynchia phoenicea* α is distributed on many reefs in the Western Indian Ocean and the Tropical Southwestern Pacific, but *F*_*ST*_ values underlined the high isolation of all sites, even separated by only ~1 km (e.g. MAY3 and MAY4), pointing toward extremely low gene flow at all spatial scales (supposing the same effective population size). *F*_*ST*_ values among sites were somewhat higher but comparable to those measured between *L. brevirostris* α populations (Postaire et al., 2017). These results highlight the role of expanses of open ocean in the metapopulation structuring of larviparous aglaopheniids. For example, in the Loyalty Islands, the *F*_*ST*_ between LOY1 and LOY2 sites on the same island was lower than the *F*_*ST*_ between LOY1 and LOY3, sites separated by open sea (*F*_*ST*_ = 0.095*** and *F*_*ST*_ = 0.145***, respectively), despite similar distances between both pairs (ca. 30 km). Along contiguous reefs, such as along the West coast of Grande Terre, *F*_*ST*_ values were comparatively low over large distances, such as between GDT3 and GDT5 (*F*_*ST*_ = 0.068***, separated by 227 km). This result may be explained by a higher probability of propagules to disperse to adjacent populations along the reef over multiple generations (through stepping‐stone dispersal). However, while being a useful metric, inferring causal relationships between *F*_*ST*_ and dispersal must be made cautiously as *F*_*ST*_ values can be modified by multiple processes as selection, inbreeding, drift and spatial subdivision: models linking *F*_*ST*_ to dispersal are frequently violated in natural conditions (Whitlock & McCauley, 1999). Nevertheless, our results point out the general high divergence among all sites across the Western Indian Ocean and the Tropical Southwestern Pacific. Indeed, Jost's *D* values showed the same trend as *F*_*ST*_ (Table S2), as well as various loci failing to amplify in individuals from several sites (potentially because of null alleles). Additionally, Bayesian clustering, PCA and network analyses identified a highly geographically structured dataset, populations grouping according to islands or archipelagoes. Furthermore, private alleles were present within all sites (but with a higher frequency in the Western Indian Ocean) and the number of alleles per loci was extremely variable among sites (sometimes even monoallelic).

The population structuring described here is comparable to the pattern uncovered in the brooding Aglaopheniidae *L. brevirostris* α in the Western Indian Ocean and the Tropical Southwestern Pacific (Postaire et al., 2017). The similar, but not identical geographic coverage of the sampling, due to the absence of the considered species at some sampling sites (Postaire et al., 2016a) and the targeted sampling of *M. phoenicea* α in Mayotte, highlight that both taxa share a pattern of high geographic clustering and population isolation across the Western Indian Ocean and the Tropical Southwestern Pacific (with the exception of some admixture in the Tropical Southwestern Pacific for *M. phoenicea* α). For both aglaopheniid species, these high levels of genetic differentiation among populations might either reflect differences in current selective pressures or testify of past variations of effective population size, for example, bottlenecks or founder events, due to past climatic and geological events (formation of new islands, sea level variations). Our results are congruent with findings in several other marine species, ranging from kelps to teleost fishes, for which a high pairwise differentiation was measured when habitat patches were isolated (Alberto et al., 2010; Billot *et al*., 2003; Riginos & Nachman, 2001). Habitat continuity might thus be an important predictor of genetic connectivity of coral reef species, having important implications for marine conservation planning, but also on macroevolutionary processes.

### 4.3 Large scale isolation by distance and speciation opportunities

In this study, we detected population IBD over relatively large spatial scales (several hundreds of km, i.e., archipelago scale or higher) and between coral reefs separated by expanses of open ocean: the Mantel tests indicated that geographic distances explained almost 70% of the genetic variance detected in the whole sampling. The absence of IBD at the island scale may reflect a bias in the method measuring geographic distances rather than an absence of correlation between genetic and geographic distance at such scale. Indeed, IBD was detected in the Chesterfield Islands among nearby populations (>100 km), but not in Grande Terre in spite of populations being sometimes separated by >300 km both on the West and the East coasts of the island (Table 2). We used Euclidian distances to measure distances between sites, ignoring the presence of landmasses and the general direction of marine currents, although they are known to influence the connectivity of marine organisms (Schiavina et al., 2014; White et al., 2010). Oceanic circulation models of the studied regions are still under development and we could not meaningfully adjust our dispersal distance estimates. However, for the Western Indian Ocean where oceanic models are more developed (Pous et al., 2014; Schott, Xie, & McCreary, 2009), our findings are consistent with those of other genetic connectivity studies (Bourjea et al., 2006; Muths et al., 2011; Postaire et al., 2017; Ridgway *et al*., 2008; Ridgway & Sampayo, 2005).

Both the observed IBD pattern and the large distribution range of *M. phoenicea* α are related to its life history traits. Similar to many other hydrozoans (Gili & Hughes, 1995), this species is potentially able of completing its life cycle while rafting, as we observed fertile adult colonies attached to floating objects (BP and HM, pers. obs.). Aglaopheniidae, and hydrozoans in general, present several key features of successful rafters [reviewed in Thiel & Gutow (2005) and Thiel & Haye (2006)]: small size, food and substratum generalists, both clonal and sexual reproduction with internal fertilization, brooding, and (assumed) nearby settlement of offspring. They have thus the ability to attach, establish, and develop persistent populations on biotic or abiotic rafts, facilitating the colonization of new habitats when encountered. Punctual rafting of larviparous aglaopheniids may allow colonizing new reefs separated by expanses of open ocean, but might be not sufficient to counterbalance genetic drift in these newly formed populations. Thus, it would not impede population divergence over time, leading to each island or archipelago hosting its own (suite of) aglaopheniid species.

Considering these results, the actual number of hydrozoans species may be considerably higher than previously thought. While their rafting ability has been proposed earlier to explain the apparent global distribution of several hydrozoan morpho‐species (Cornelius, 1981, 1992), inferring distribution ranges of hydrozoans species based on morphology alone might be erroneous as morpho‐species that comprise multiple cryptic species and allopatric lineages are common (e.g., Leclère et al., 2007; Moura et al., 2012; Postaire et al., 2016a). In *M. phoenicea* α, previous phylogeographic analyses using mitochondrial and nuclear markers revealed two divergent lineages, one occurring in the Western Indian Ocean and the other in the Tropical Southwestern Pacific (Postaire et al., 2016a). Species delimitation methods based on DNA classified both lineages as robust hypotheses of allopatric species, but as they were never found in sympatry, their biological species status could not be confirmed. However, the present analyses supported the high divergence between the Western Indian Ocean and the Tropical Southwestern Pacific with constantly high *F*_*ST*_ and Jost's *D* values between sites. In addition, the hierarchical approach used in the clustering and network analyses highlighted the importance of geography in the population structuring of *M. phoenicea* α across the whole studied area. Thus, *M. phoenicea* α from the Western Indian Ocean and the Tropical Southwestern Pacific represent two lineages situated in the gray zone of the speciation process (De Queiroz, 2007; Pante et al., 2015) or already two distinct species.

## 5 CONCLUSIONS AND IMPLICATIONS FOR MARINE BIODIVERSITY CONSERVATION

Our study revealed that *Macrorhynchia phoenicea* α is composed of multiple, highly genetically isolated metapopulations, with low genetic diversity and high consanguinity (or traces of population functioning mainly via asexual reproduction). The simplest explanation for the observed genetic structuring and low connectivity is larviparity: limited planktonic dispersal capacity induces small effective population size by reducing gene flow between populations, accelerating genetic drift. This reproductive strategy combined with the inferred capacity to successfully disperse through rafting can account for their apparent extended distribution but these traits also enhance speciation opportunities. From an evolutionary point of view, each island hosts a species (*sensu* Samadi & Barberousse, 2006) and our study highlights the preeminent role of allopatrism and vicariance in the diversification of coastal brooding species (Paulay & Meyer, 2002). Rather than real cosmopolitan species, hydrozoans and many other marine organisms are likely mosaics of morphologically similar independent metapopulations, or even species (depending on the criterion used), and thus should be studied accordingly (Pante et al., 2015). These results highlight that speciation in the sea can occur at small spatial scales, contributing to the accumulation of species in marine biodiversity hotspots.

The observed geographic structuring does not correspond to defined biogeographic ecoregions (Spalding et al., 2007), exemplified by the Western and Northern Madagascar ecoregion comprising three clusters and New Caledonia, at least four (i.e., the Chesterfield Islands, West and East coasts of Grande Terre, and the Loyalty Islands). Similar discrepancies have been observed in several organisms from the Western Indian Ocean, such as scleractinians (Ridgway & Sampayo, 2005; Ridgway et al., 2008), coastal fishes (Muths et al., 2011), marine turtles (Bourjea et al., 2006), and hydrozoans (Postaire et al., 2017), highlighting the disjunction between the northern and southern parts of the Mozambique Channel and the isolation of Juan de Nova Island, probably due to the presence of oceanic gyres. Our results underline that the hierarchical three‐level classification (i.e., realm, province, and ecoregions) proposed by Spalding et al. (2007) is too coarse to encompass the genetic diversity of larviparous hydrozoans and potentially many other marine species. For marine brooding organisms with low PLDs, each island/archipelago could potentially represent an evolutionary hotspot (Hoareau *et al*., 2013; Vandergast *et al*., 2008), underlining the need of a network of marine protected areas to ensure the conservation of marine organisms as well as the maintenance of evolutionary mechanisms across oceans, rather than delimiting a limited number of extended marine sanctuaries.

## CONFLICT OF INTEREST

None declared.

## DATA ACCESSIBILITY

Microsatellite genotypes are available on Dryad DOI: 10.5061/dryad.cb0b8.

## AUTHOR CONTRIBUTIONS

B.P., J.H.B., and H.M. designed the research. B.P., P.G., M.P., and H.M. generated the data. B.P., P.G., and H.M. analyzed and interpreted the data. B.P., P.G., J.H.B., M.P., and H.M. wrote the manuscript.
