# Supplementary figures and images for: Population differentiation or species formation across the Indian and the Pacific Oceans? An example from the brooding marine hydrozoan Macrorhynchia phoenicea
Source: Ecol Evol. 2017 Sep 6;7(20):8170–86. doi: 10.1002/ece3.3236 (PMC5648676; doi:10.1002/ece3.3236)

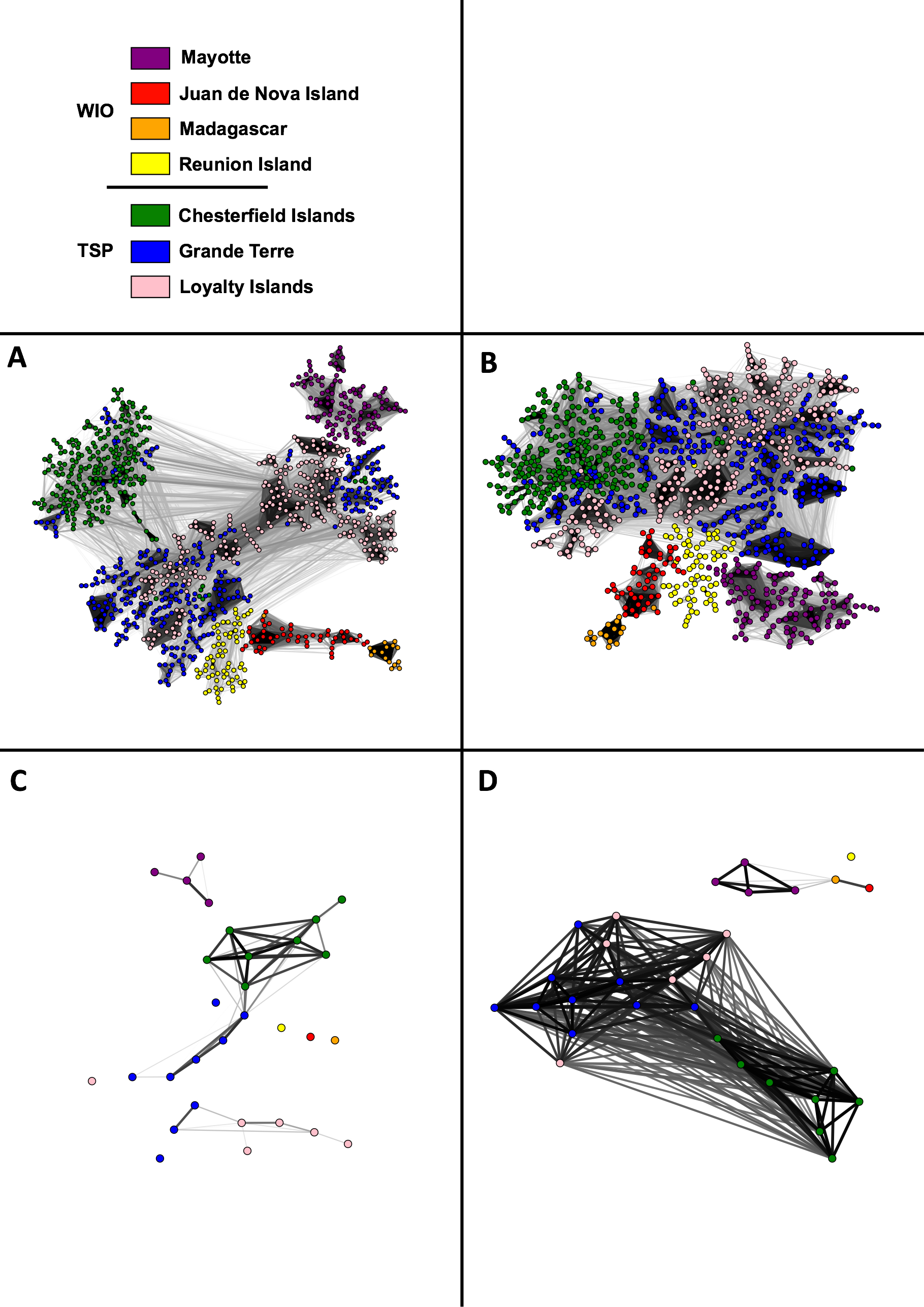

Supplement: Supplementary file 1 [file ECE3-7-8170-s001.jpg]

Western Indian Ocean

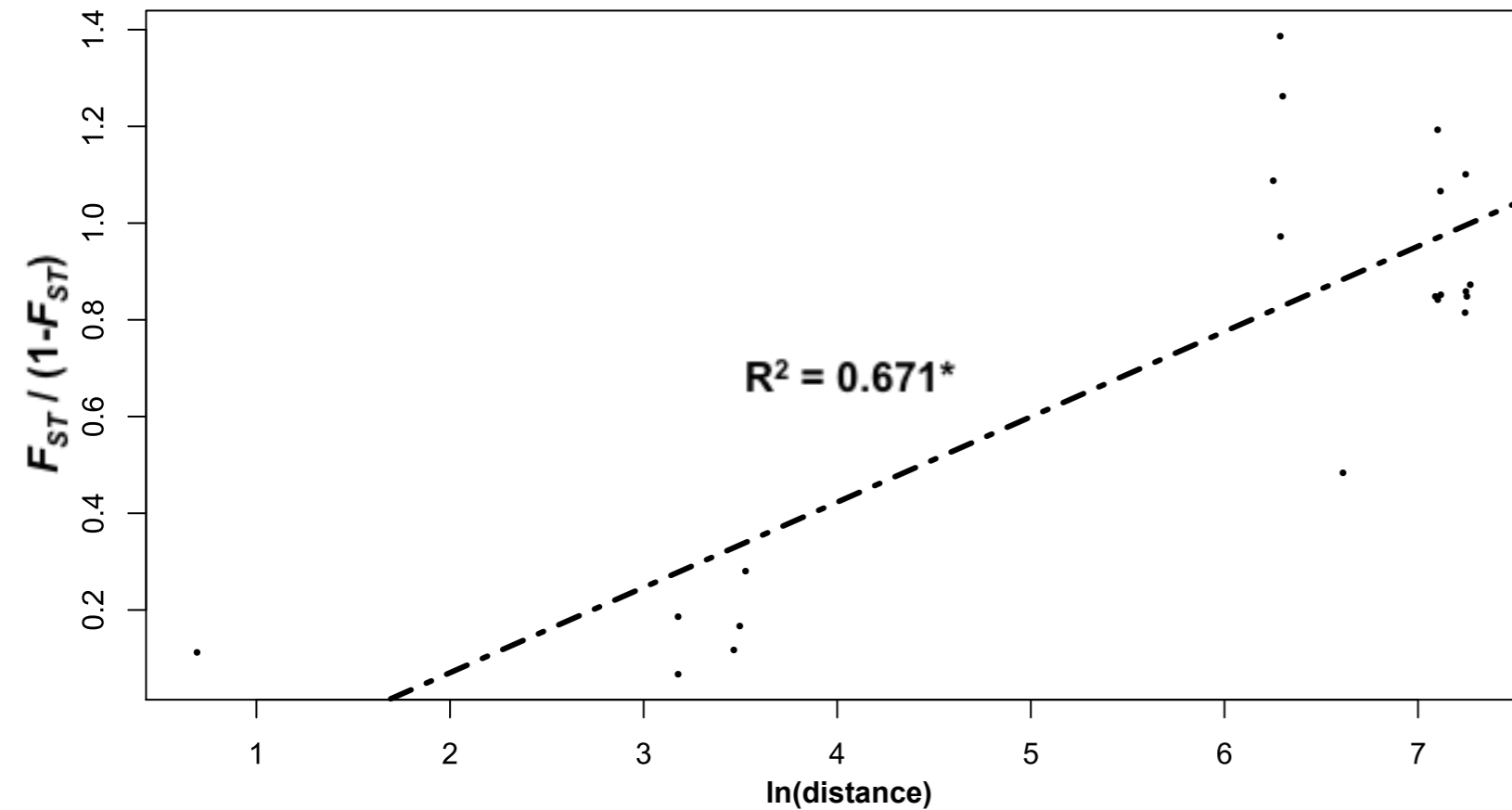

Tropical Southwestern Pacific Ocean

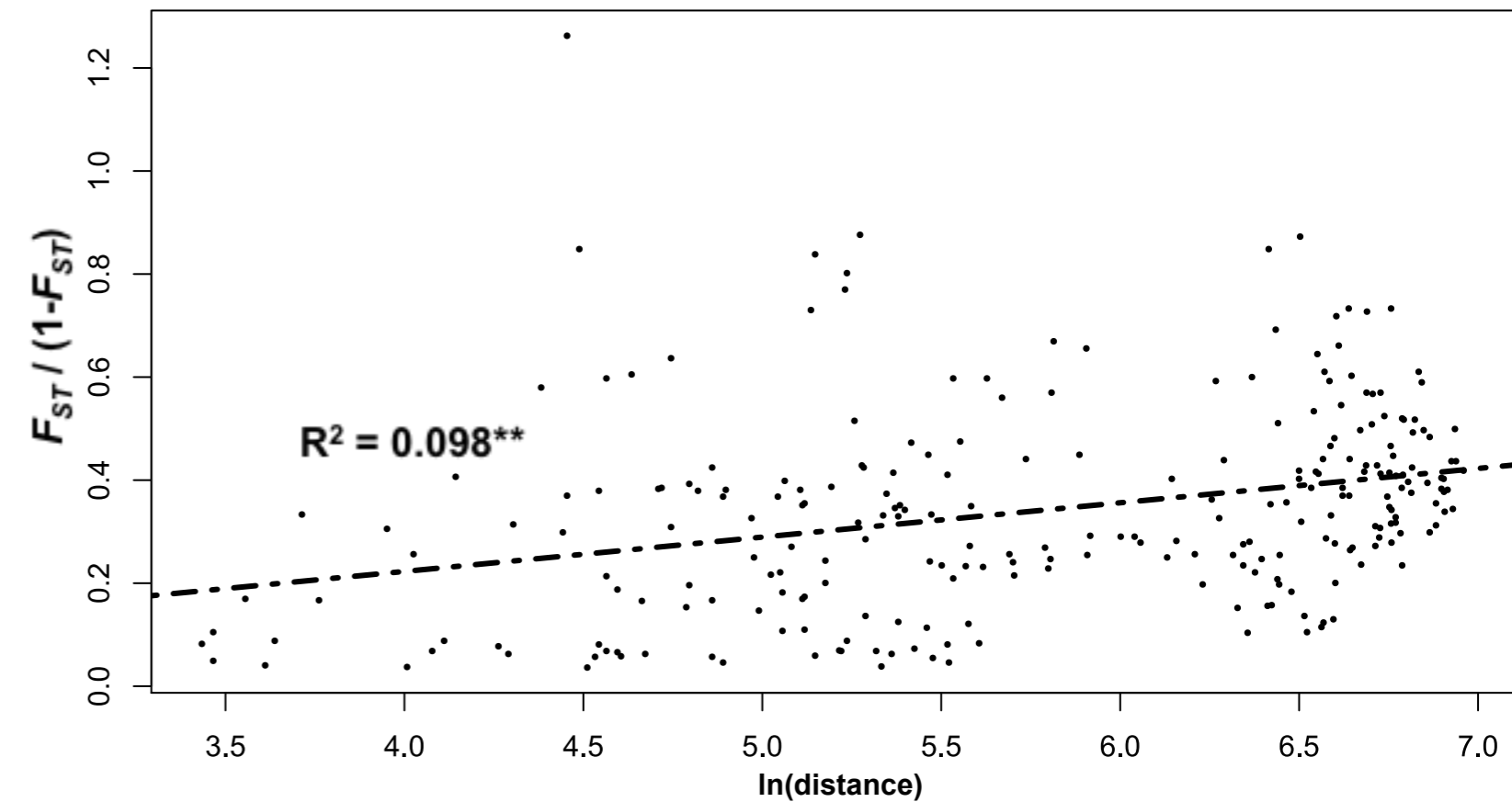

Supplement: Supplementary file 2 [file ECE3-7-8170-s002.pdf]
